# Making a Home

**Published:** 1875-12

**Authors:** J. Hall


### MAKING A HOME.

BY REV. DR. J. HALL.

Nothing in the arts, or in manufactures, is so important to the community, as the making of a home. A volume may well be written on the subject—will not some competent pen undertake it?—and only a few hints can be dropped in this article, which thoughtful readers, looking ahead, will please ponder, forgiving the author if he fail to hit their individual difficulty, or commend all their individual merits. on this line.

Usually it takes two persons to make a home. We cannot conceive of the thing being done with fewer "hands;" and if one of the "hands;" has been sought in manly, pure, reverent love, and given after thought and care, and with genuine womanly devotion, all the better. We do not mean to say that parent and child, brother and sister, teacher and pupils may not have a home; for they may; but we speak of the best kind of ordinary home. If Mr. Smith lives in a "hall bedroom" at night, and in the "office" in day.light, he has a boarding-house," and a "den," but—and well he knows it poor fellow! he has no home. He had better be there, to be sure, than in the police-station, or many other places, but he is not in the most favorable conditions for life, happiness and usefulness.

Two persons going into partnership for the making of a home need to understand one another. This understanding should be founded on some acquaintance and friendly intercourse. If a man sees a nice girl at a restaurant, proposes, and is accepted, in some measure because the girl is tired of the restaurant, he need not wonder if there be developed such diversity of view and taste as will preclude cordial co-operation. If a young girl is caught by the whiskers—dyed, perhaps—of a strange gentleman who complements her on her singing in the Bulbul Choral Company, and invites his homage, she need not wonder if there be a good many things about him as unreal as the whiskers, of which she only becomes aware when married. And marriage—public, honorable, deliberate marriage—is essential to a right partnership of this kind. Usually the approval of relatives is needful. Girls who believe that men are going to take them to a paradise, without a wedding ring and a marriage certificate, pay a fearful penalty for credulity and folly.

The partnership formed, if possible a separate nest should be found. It is a good rule to let young men and women, when married, "forsake father and mother and cleave unto" each other. They do best when left to themselves, nobody to meddle with their joys, or with the little mutual adjustment of temper, feeling, and preference, inevitable to married life Dick will stand a quick word from Dolly, who will kiss it away by and by; but if Dolly's mother becomes a party to the misunderstanding, she cannot kiss the trouble out of sight. So Dick and Dolly had better be by themselves, even if the domicile be lowly, in their earliest married life—in a house of their own, if possible; but if this cannot be, in their own "apartments."

And the house or apartments should always be within their means, carefully calculated. A man who feels, as he puts the latch-key into his own door, that it costs him a deal too much, carries about a thorn in his mind. And when he says, "Jenny, dear! we are living at too high a rate," Jenny is apt to take it as a reflection on her housekeeping, or dressing, or something of that sort, and to recur to something that Harry could do without, and great troubles ensue. Rent is a terrible, malevolent power. You rest, it goes on; you work, it goes on. You wake or sleep, are busy or idle, but like the motion of the earth, it is incessant. Once in for it, and you can no more save on that line than you can comprehend the currency question, or pay your debts by giving your notes. The rent, if one does not own his house, should always be kept well in hand.

Nor should any conspicuous article be allowed inside the door, that puts to shame the rest of the movables. A velvet carpet, blazing in all the hues of the Orient, beggars the plain sofa and homely chairs. The table looks like a four legged impertinence upon it. Mrs. G.'s eye is offended hourly; so are her visitors'. They drop hints. The depreciated furniture has to take itself off. You get new articles—and some debt—and some anxiety—and some more demands—for the fine furniture reproaches the house; and the vexation is endless. Get things of a piece with your means, and of a piece with one another. Don't be afraid to say, "We are not yet rich;" and if the plain chairs and tables say it to your friends, why let them. Is it not written down in the "Declaration of Independence" that God hath made all men free and equal? Will twelve feet square of a mirror make any real difference in the actual sum of your home-life one way or another? If Mr. and Mrs. Skyhigh do not visit you because you lack the mirror, why let them stay at home and contemplate their own. You and Mary—God bless her!—many a time said you "were all the world" to each other. You can do without the mirror and Mrs. Skyhigh; till the one can be fairly afforded, and the other finds that you are just as good as she is.

And as the years roll on, and the home has in it many sons and daughters, it is wise and well to give each, if possible, a place of his or her own. It may be very small, boarded off, a mere "cupboard of a place;" but no matter. It is good for each, when the nursery is deserted, to have a room of his own. The Bible is wise always: "Enter into thy closet." A "dormitory" is a difficult place in which to be good. Let the young ones, if it be possible, have their little nooks, and be responsible for keeping them neat, sweet, and tidy. We were not meant to "herd together," but that each life should have some sacred sweetness of its own about it.

And as far as possible, there should be gathered in the home the objects that please the eye, and get hold of memory. They need not to be costly, nor many. A bewildering crowd of things—such as one may see now a days in parlors, hideously ugly, but fashionable, is an abomination. Prints, flowers,pictures, autumn leaves deftly grouped together, little mementoes that speak of love, recollection, considerateness—these are of more value than a thousand dollars worth of imported trash that is made " the thing " at Christmas and New Year's day, and which you must buy because " there's positively nothing else to be got."

Let the children's hearts and minds grow in the balmy, healthy air of homes like these, and when long years hence, they are far away, have become gray-haired men or anxious mothers, and their parents are here no more, they will recall the quiet, dear, homely delights that cost little, and meant, oh! so much; they will connect them with you—your care, your love, your lessons—and the memory will be to them a benediction.

